# Processed Dietary Patterns during Pregnancy Are Associated with Low Birth Weight at Term among Women of Advanced and Non-Advanced Age

**DOI:** 10.3390/nu14163429

**Published:** 2022-08-20

**Authors:** Tzu-Ling Chen, Su-Fen Cheng, Meei-Ling Gau, Li-Li Lin

**Affiliations:** 1 Department of Nurse-Midwifery and Women Health, National Taipei University of Nursing and Health Sciences, Taipei 11221, Taiwan; 2 Department of Allied Health Education and Digital Learning, National Taipei University of Nursing and Health Sciences, Taipei 11221, Taiwan; 3 Obstetrics and Gynecology ward, MacKay Memorial Hospital, New Taipei City 251031, Taiwan

**Keywords:** dietary pattern, low birth weight, maternal age, gestational weight gain, pre-pregnancy BMI

## Abstract

Inappropriate dietary intake during pregnancy is a key factor in low birth weight (LBW). This study compares LBW between healthy and processed dietary patterns by focusing on women of advanced maternal age. A cross-sectional survey was conducted with 327 postpartum women in Taiwan. The participants were assigned to two groups according to their age (≥35 years, n = 151; and 20–34 years, n = 176). An online questionnaire asked women how often they consumed 27 food items during their pregnancy. The prevalence of LBW was higher in the processed dietary pattern (79.3%) than in the healthy pattern (13.78%, *p* < 0.001). LBW was positively correlated with advanced maternal age (≥35 ages), low pre-pregnancy weight (BMI less than 18.5 kg/m^2^), insufficient gestational weight gain (GWG), and processed dietary patterns. Older mothers were 5.8 times more likely to have infants with LBW (odds ratio = 5.8; 95% confidence interval 2.0–16.6). A processed dietary pattern was 9.4 times more likely to result in LBW. Insufficient GWG was significantly positively associated with LBW (OR = 4.0; 95%CI 1.4–11.6). Maternal diet during pregnancy is an important modifiable factor for LBW. Prenatal advice should emphasize optimal nutrition, especially in older and underweight women.

## 1. Introduction

A processed dietary pattern refers to behaviors that favor savory, fried, and processed food products that contain high levels of saturated fat, free sugars, sodium, and trans-fats, such as baked goods, savory snacks, sauces, dressings, and condiments [1], which have a negative effect on the health of the individual and can also adversely affect the birth weight of infants [2]. In Taiwan, as in other countries, socioeconomic changes are related to changes in dietary patterns [3] that are associated with a reduction in daily calorie consumption, higher consumption of unsaturated fats, added sugar, and processed foods, and lower consumption of fruits and vegetables [4]. This dietary pattern is a matter of concern at all stages of life and especially during pregnancy, where it is associated with poor maternal health and compromises fetal growth and development [5,6]. The lack of or excess nutrients during pregnancy can lead to morbid complications for both the mother and fetus [7] and can impact a child’s health at later stages of life.

Studies have shown an association between maternal dietary patterns during pregnancy and infant birth weight; however, the findings remain inconsistent. A study in northern Ghana has revealed that healthy eating patterns during pregnancy (vegetables, fruits, cereals, legumes, roots and tubers, and few high sugar/energy snacks) have a protective effect against low birth weight [5]. 

A recent systematic review of observational studies on 167,507 women suggested that an unhealthy diet (e.g., refined grains, processed meat, and sources of saturated fat and sugar) during pregnancy causes inflammation in physiological systems. Such inflammation further decreases placental fetal blood flow, which is associated with lower birth weight and increased chances of prematurity [8]. The composition of the maternal diet is critical during the early stages of pregnancy, specifically for the development and differentiation of organs. Additionally, during the later stages, poor maternal nutrition can detrimentally impact the fetal growth rate and brain development [9]. However, research on the relationship between dietary patterns during pregnancy and fetal growth has been limited to Western countries. Associations between fetal development and maternal diet that are assessed based on dietary patterns and diets during pregnancy widely vary across cultures. Compared to Western diets, Eastern diets contain less cheese, alcohol, fruits and vegetables, and potatoes as staple foods [8,9,10].

In the East, pregnant women tend to include more fruit in their diet during pregnancy. To avoid neonatal allergies, pregnant women also tend to lower their intake of vegetables, eggs, fish, and shellfish during pregnancy [11]. Since dietary habits are often induced by culture and by the type of food available, investigating the characteristics of diets and practices in a study population may provide a basis for future interventions to improve maternal and infant nutrition.

With the development of society and changes in people’s views on procreation (assisted reproductive technology, higher levels of education and participation in the workforce by women, lower marriage rates, and an increase in double-income partnerships with no kids), the global trend of delaying childbirth is increasing [12,13]. In Taiwan, the mean maternal age and the proportion of older pregnant women (≥35 years) are increasing. The number of primiparas over the age of 35 in Taiwan has increased from 9.4% in 1998 to 23.0% in 2019, and the number of primiparas has increased by 13.6% over the past ten years [4]. In 2015, of the 20.5 million infants born globally, an estimated 14.6% suffered from low birth weight (LBW) [14]. According to a systematic review and meta-analysis, women ≥ 35 years are at a higher risk of giving birth to LBW infants (<2500 g) compared to women < 35 years [15]. Studies in China have shown that among women > 34 years old, their infants’ birth weight decreased by 0.824 g for every additional year added to the mother’s age [16]. LBW (BW < 2500 g) significantly increases the risk of perinatal mortality and morbidity [16,17]. Few studies have examined processed dietary practices and their effects on LBW at term between women in different age cohorts. Therefore, the main aim of this study was to investigate maternal dietary patterns in relation to birth weight, specifically the effect of processed dietary patterns and their association with LBW irrespective of maternal age.

## 2. Participants, Ethics, and Method

### 2.1. Sample Size Estimation

Based on the results of a Chinese study [11], we hypothesized that the effect size of differences in infant birth weight between women of advanced and non-advanced maternal age is 0.5 [18] (p. 605). Using G power 3.1.7, the required sample size was calculated as 80 in each group for an alpha error of 0.5, two-sided alpha of 0.05, and power of 0.8. Finally, we recruited 176 women (advanced) and 151 women (non-advanced) for a total of 327 participants in the present study, which may prevent missing significant predictive variables during the analysis.

### 2.2. Design and Participants

This study was a cross-sectional study. The inclusion criteria were adult women (20 years of age) and primipara without preterm birth (<37 weeks), severe maternal conditions (including gestational diabetes, pregnancy-induced hypertension, and pre-eclampsia), presence of anemia, COVID-19, other systemic and reproductive tract infections, or thyroid function or neonatal complications. Participants with no available adherence to prenatal care based on Taiwan’s health insurance and who used supplements, tobacco, drugs, or alcohol during gestation were excluded. We completed interviews for 3–5 days postpartum. The first author assessed the eligibility of the pregnant women (clinical data collected from the maternal health booklet during Taiwan’s prenatal medical care) recruited [19] from January 2022 to May 2022. A consecutive sample was solicited from a hospital obstetrics outpatient center. The principal investigator (PI) contacted each eligible participant at their third-trimester prenatal check-up, explained the study’s purpose, and asked whether they would like to participate. Those who agreed to participate were asked to sign a consent form and give their e-mail address to the PI. The data were collected on postpartum days 3–5 through an online questionnaire. Of the 332 women approached, 5 refused to participate in the study. In total, 327 postpartum women were enrolled, of whom 176 were aged 35 years or older (advanced age group) and 151 were aged 20–34 years (non-advanced age group). There were no significant differences in mean gestation weeks between the two groups.

### 2.3. Measurements

We designed the study tools based on a literature review and clinical observations. The study tools included: (1) a self-designed structured questionnaire, which comprised demographic information (maternal age and the family’s socioeconomic status), pre-pregnancy BMI, and categories of weight gain during pregnancy. Family socioeconomic status was classified into high (41–55), middle (30–40), and low (11–29) groups based on the Hollingshead [20] 2-factor index of social position, which was adapted to Taiwan by Lindsay [21]. Maternal body weight and height were self-reported, and participants’ pre-pregnancy body mass index (BMI) was calculated by the researcher. BMI cut-offs were based on Centers for Disease Control (CDC) guidelines [22] and used to classify the participants as follows: underweight (<18.5 kg/m^2^), normal weight (18.5–24.9 kg/m^2^), overweight (25.0–29.9 kg/m^2^), and obese (≥30 kg/m^2^). Weight gain during pregnancy was calculated using data from participants’ antennal medical records. The categories of weight gain during pregnancy were based on pre-pregnancy BMI and Institute of Medicine (IOM) recommendations [23]. Weight gain during pregnancy, defined as gestational weight gain (GWG), was described as “insufficient” if the weight was below the IOM recommendation, “adequate” if the weight gain was within the recommendation, and “excessive” if the weight gain was above the recommendation. (2) Infant characteristics included childbirth mode, gestational age (calculated based on the date of the first day of the last menstrual period), weight, and sex. Information on birth weight and sex was extracted from medical certificates that were provided by clinical pediatricians. Low infant birth weight (LBW) was taken as <2500 g based on the World Health Organization’s (WHO) classification [24]. (3) Dietary food consumption patterns during pregnancy were assessed using a food frequency questionnaire (FFQ). Taiwan’s food culture is complex and diverse, and the culture of eating out is prevalent. Therefore, in this study, 27 food items that are representative of the usual Taiwanese diet were selected and included in the questionnaire. Additionally, the questionnaire was designed to help capture foods with small variations in respondents’ intake (such as rice and meat), which can be used to evaluate their overall dietary content and reduce the possibility of duplicate entries [25]. Athanasiadou et al. demonstrated that collecting dietary information for the last month can help avoid bias and increase the chances of respondents accurately recalling their dietary intake. Therefore, to reduce memory bias, the FFQ assessed the participants’ dietary intake during the month prior to their delivery [26]. In this study, participants were asked about individual foods in broad categories: rice/noodles; bread/pasta/cereals; eggs; chicken, beef, or mutton; seafood; vegetables; pickled vegetables; snacks, cakes, and biscuits; and drinks, fried food, and fast foods. The questions were presented in the following manner: “How often do you on average have of the following foods since you became pregnant/when you were pregnant.” The choices were: (1) not at all, (2) once a month, (3) 2–3 times a month, (4) once a week, (5) 2–3 times a week, (6) 4–6 times a week, (7) once a day, and (8) 2–3 times a day. The FFQ content validity of the self-designed structured questionnaire was reviewed by six experts, which included two nutritionists, one obstetrician, two nursing professors, and one deputy head nurse in a postpartum ward. The experts rated the appropriateness of the questions on a 5-point Likert scale (from 1 to 5, very inadequate to very adequate) and provided feedback on revising questions that received scores of <3. The content validity index was 0.95; that is, 95% of the questions received a score of 3.

### 2.4. Data Analysis

#### 2.4.1. Dietary Patterns

The FFQ included 27 general food items. First, an exploratory factor analysis was conducted to generate dietary patterns. When deriving dietary patterns, it is a common practice to pre-group food items before calculating the principal components through an optimal weighted linear combination of food groups based on their correlation. Among all principal components, only a few that explain the most variation are retained for subsequent analysis [27]. The analysis to determine the characteristics of the dietary patterns was carried out using all 27 food items in the questionnaire. Varimax rotation of the factors was employed, and a graphic scree plot was used to define the extracted (retained) factors. Dietary patterns can be derived a priori, where foods are classified into patterns based on their known functions and effects on health, or a posteriori. We used the percentage of variance explained by each factor and a scree plot to determine the number of factors. Food groups were descriptive of the dietary pattern if their factor loadings had a magnitude of 0.4 or greater [28,29]. Factor 1, which had higher scores for fresh vegetables, fruits, rice/noodles, chicken, meat, or mutton, and dairy foods/milk and negative scores for high-sugar snacks (such as sweets, cookies, and chocolate), was described as the “healthy” dietary pattern. The healthy eating pattern described by the Taiwan Health Promotion Administration [19] includes vegetables, fruits, whole grains, low-fat dairy, or lean protein food (i.e., seafood, lean meat/poultry, eggs, legumes, nuts/seeds, and soy products). Factor 2, which had a higher loading for high-energy snacks and lower scores for fresh vegetables, fruits, rice/noodles, cereal, and meat, was described as the “processed” dietary pattern. Prior to the analysis, the Kaiser–Meyer–Olkin measure of sample adequacy, which is a prerequisite for factor analysis, was performed and found to be in an acceptable range (0.89). A Bartlett’s test of sphericity was also performed and showed no evidence of identity of the correlation matrix in the data, and the data set was considered appropriate for factor analysis (Prob > chi2 = 0.0000). The internal consistency of each factor was evaluated using Cronbach’s alpha, where “healthy pattern” was 0.88 and “processed pattern” was 0.89. 

Second, the consumption frequency of each food group was calculated by summing the frequency of each item within the food group. For each participant, the contribution (%) of every food group was calculated by dividing the consumption frequency of each food group by the total consumption frequency across all food groups [30]. The weekly percentage intake of the 27 food types was taken into account to construct the dietary patterns. A cluster analysis was performed using the k-means procedure in data analysis using R software (4.1.2). The latent profile analysis (LPA) method was applied to classify participants into a predetermined number of mutually exclusive groups by comparing Euclidean distances between each participant and each cluster center in an interactive process until no further changes occurred. The LPA explored the dietary frequencies of each food that create mutually exclusive groups of people with relatively homogeneous food consumption patterns; individuals could only belong to one identified pattern. A combination of the lowest Bayesian information criterion (BIC) and the lowest parametric bootstrapped likelihood ratio test (BLRT) [31] helped to determine which model provided the best fit. Based on the fit index for BIC and BLRT derived from the LPA results, two group SCs (BIC = 34574.87, *p* = 0.001) were identified. Based on the fit index for BIC and BLRT derived from the LPA results, two SCs (BIC = 34728.93, *p* = 0.001) were obtained (see Table 1). Third, the statistical methods in our analysis used factor analysis to categorize foods that are usually eaten together into two groups. The identified dietary patterns were as follows: “healthy” 79.5% and “processed” 20.5%. 

#### 2.4.2. Characteristics of the Women in the Two Age Groups

Descriptive analyses were performed by calculating frequencies and percentages for the categorical variables. Chi-squared tests or Fisher’s exact tests were used to examine differences in the characteristics of the women in the two age groups. Fisher’s exact tests were used when at least one cell of the table had an expected count smaller than 5. A logistic regression model was used to identify factors related to low infant birth weight at term and to examine the net effect of advanced maternal age on low infant birth weight, given that other variables in the model were adjusted for. At first, we started with a model for infant birth weight with only advanced age as the independent variable. Then, the study variables were entered into the logistic regression model. Logistic regression modeling was used to examine the association between dietary patterns and LBW. Initially, dietary patterns were entered into the logistic regression. Then, the researchers dropped the least significant (*p* < 0.05). Lastly, all remaining significant variables were re-entered into the multiple logistic regression at the same time to yield the final model. The overall fit of the final logistic regression model was assessed by the Hosmer–Lemeshow goodness-of-fit test with a *p*-value of > 0.05, indicating an acceptable model fit [32]. The data analysis was performed using Statistical Package for the Social Sciences (SPSS) for Windows version 22.0 (SPSS Inc, Chicago, IL, USA).

## 3. Results

Of the 327 women recruited, 176 (53.8%) were 35 years or older (advanced age group; mean age 40.4 ± 2.8; range 35–45 years), and 151 (46.2%) were aged 20–34 years (non-advanced age; mean age 31.6 ± 3.1; range 20–34 years). The mean birth weight was 3115.5 ± 409.3 g (range 1800–4300 g). Among the participants, 29 (8.9%) had LBW infants. The characteristics of the participants are compared in Table 2. Our participants were predominantly college or university educated (95.1%). The majority’s pre-pregnancy BMI was normal (71.3%), and 30.0% of the respondents’ childbirth mode was by cesarean section. Women in the advanced age group were more likely to have full-time employment (60.8%) and a high socioeconomic status (52.3%) than those in the non-advanced age group (48.3%, *p* = 0.02; 40.4%, *p* = 0.08). The mean GWG of the participants was 11.1 ± 4.8 kg (range 0–35 kg). Approximately 46.2% of the women’s GWG was “insufficient,” 41.9% was “adequate,” and 11.9% was “excessive.” Women in the advanced age group (age ≥ 35 years) were significantly associated with higher instances of LBW (13.1%) than the younger women (20–34 years, 4.0%).

Most participants (81.0%) showed a “healthy” dietary pattern during pregnancy. Women ≥ 35 years had lower incidences of “processed” dietary patterns (17.6%) than women < 35 years (20.5%, *p* = 0.50). The dietary patterns by maternal characteristics are compared in Table 3. Those with a higher socioeconomic status tended to have a healthy dietary pattern rather than a processed dietary pattern (48.7% vs. 38.7%, *p* = 0.33). Women’s work status as full-time employees was weakly significant, and they were more likely to have a healthy pattern than a processed pattern (57.4% versus 45.2%, *p* = 0.07). A “processed pattern” was significantly associated with insufficient GWG (54.8%) than a “healthy pattern” (44.2%). A processed dietary pattern was more significantly associated with LBW than a healthy pattern (25.8% versus 4.9 %, *p* < 0.001).

We merged pre-pregnancy BMIs for overweight and obesity into one group (overweight and obesity). Crude and adjusted ORs from the logistic regression are presented in Table 4. Of all of the variables included (as in the Measurements section), advanced maternal age, underweight pre-pregnancy BMI, insufficient GWG, and a processed dietary pattern were significantly associated with LBW (crude OR = 3.3–6.7). We adjusted OR for dietary patterns considering maternal age, pre-pregnancy BMI, GWG, and childbirth weeks in the final model. The final multiple logistic regression results showed that advanced age (≥35 years) women (OR = 5.8; 95%CI 2.0–16.6) and a processed dietary pattern (OR = 9.4; 95%CI 3.7–23.6) had higher odds for LBW at term (<2500 g) than those in the younger age group (20–34 years), and a processed dietary pattern was significantly positively associated with LBW at term (<2500 g). Underweight pre-pregnancy BMI had higher odds of LBW at term than normal pre-pregnancy BMI (OR = 6.8; 95%CI 2.3–20.1). Insufficient GWG was significantly positively associated with LBW at term compared to normal GWG (OR = 4.0; 95%CI 1.4–11.6). This model yielded a Cox and Snell r^2^ = 0.15, Nagelkerke r^2^ = 0.32, and a Hosmer–Lemeshow goodness-of-fit χ^2^ = 13.26, df = 8, *p* = 0.10.

## 4. Discussion

We defined two dietary patterns and demonstrated how mothers’ dietary patterns are associated with LBW. Processed diets were positively associated with LBW. A “processed” dietary pattern included fried meat, processed meat and poultry, refined grains, sweets, desserts, fast food, snack foods, soda, and sweetened beverages, whereas a “healthy” dietary pattern included fresh fruit, vegetables, whole grains, fish and seafood, legumes, poultry, nuts, seeds, and non-processed meat. 

In this study, a healthy dietary pattern offered greater protection against LBW. Similarly, a study in northern Ghana involving pregnant women reported two patterns of food consumption: healthy (local preparations containing maize flour and maize meal, yams, fruits, vegetables, meat, and eggs) and unhealthy (sweetened drinks, ice cream, chocolate drinks, and soda); the healthy pattern was also associated with lower odds for LBW [5]. Chia et al. [8] conducted a systematic review and meta-analysis and showed that unhealthy dietary patterns (denoted “processed conscious”) characterized by high intakes of refined grains, processed meat, and foods high in saturated oils or sugar were associated with LBW. A recent study also showed that processed foods associated with Western diets are similarly characterized by processed meat, potatoes, sweetened snacks, and saturated fats [33]. Our participants’ food quality in the processed dietary pattern was also characterized by the highest amounts of deep-fried food, processed foods, and foods with added sugar, as well as the highest amount of dietary sugars, all of which could be positively associated with LBW. We found that maternal exposure to high-quality diets characterized by a high intake of pickled vegetables, juice, sugar-sweetened beverages and fruit juice, and processed meats were also at risk of having LBW infants. In addition, there is evidence of a secondhand sugar effect, namely, that maternal sugar consumption may increase pregnancy complications and decrease infant birth weight [34]. We hypothesized that a pro-inflammatory dietary pattern, such as a diet high in sugar, refined flour, saturated fats, and red and processed meats, can trigger inflammation [35]. During pregnancy, there is a physiological increase in systemic inflammation and oxidative stress. Excessive pro-inflammatory production of free radicals can act as a stressor that is associated with low antioxidant defense and can thereby negatively impact the placenta and subsequently increase the risk of adverse birth outcomes [36], including LBW. A “healthy” dietary pattern was mainly characterized by fresh pork, seafood, poultry, vegetables, and fruit, while an “unhealthy diet” entailed a high intake of refined grains, processed meats, and food high in saturated fats or sugar. Therefore, the dietary exposure group differed somewhat from the groups in our study; for example, sugary drinks, juices, and sodas were categorized under the “processed” pattern in our study. Conversely, the food items were categorized under the “healthier dietary pattern” in a Mexican study of pregnant women [10]. Food intake and dietary patterns can differ even between countries as geographically close as China and Taiwan; for instance, consumption of desserts and soup is higher in Cantonese China. Similarly, in Lu et al.’s [11] China-based study, they found that a traditional Cantonese diet high in cereals, eggs, and Cantonese soup and a diet high in fruits, nuts, and Cantonese desserts (Chinese buns, egg tarts, rice cakes, and mooncakes) could be associated with higher birth weight, while a varied diet might be associated with higher birth weight and a decreased risk of having a baby of low gestational age. A Norwegian study showed that a “traditional” diet characterized by a high intake of lean fish, fish products, boiled potatoes, and cooked vegetables was associated with lower birth weight. This pattern was closer to that observed in our “healthy” group, but with a high content of dark bread, low-fat milk, and lean fish [37]. Our study’s findings suggest that pregnant women should avoid imbalanced—excessive or deficient—food consumption. In other words, healthy living habits with a varied dietary intake, specifically qualitatively balanced nutrients and energy that meet pregnancy-related nutritional needs, aid in the satisfactory adaptation of maternal physiology and the adequate growth and development of the fetus [2]. We observed a significant negative association between maternal age and LBW when the maternal age was over 35, which is consistent with the findings of a previous study on women in China [16]. However, the impact of maternal age on infant birth weight remains unclear. Relevant research has demonstrated that epigenetic DNA reprogramming that occurs during the development of the embryo or fetus is a major risk factor that can cause intrauterine exposure to maternal age-related obstetric complications [38]. Studies have widely acknowledged that mitochondria are maternally derived. Therefore, mitochondrial DNA is subject to a greater risk of mutations that are associated with age, given its inability to repair DNA, and this, in turn, affects infant birth weight. Additionally, the prevalence of healthy dietary patterns tends to be higher in older age groups. Our results demonstrate that a healthy dietary pattern during pregnancy is conducive to adequate infant weight and size at birth; this may have been confounded by pregnant women in the older age group aiming to reduce the risk of complications [38]. This possibility cannot be ruled out despite additional adjustments for certain pregnancy-related diseases in our study. 

We found that compared to normal pre-pregnancy BMI, underweight was associated with LBW at term and with a higher odds ratio. A study in Japan demonstrated that maternal pre-pregnancy BMI less than 18.5 kg/m^2^ increased the risk of preterm births and infants who are small for their gestational age [38]. We found that compared to normal pre-pregnancy BMI, “underweight” was independently associated with LBW at term with a higher odds ratio. A study in Japan demonstrated that maternal pre-pregnancy BMI less than 18.5 kg/m^2^ increased the risk of preterm births and infants who are small for their gestational age [39]. To prevent preterm and low birthweight infants, particularly among older women, education about appropriate nutrition is key to maintaining a normal BMI. In addition, balanced weight and adequate vitamin and mineral intake are vital for ensuring that pregnant women are healthy and for reducing risks to newborns [2,36,40]. In this sense, maintaining healthy eating habits is a requirement both before and during gestation. Thus, to improve adherence to healthy dietary practices and reduce the risk of LBW, healthcare professionals may need to discuss the importance of maintaining pre-pregnancy body weight and following the Taiwan Health Promotion Administration [19] food intake guidelines with pregnant women.

GWG was negatively associated with LBW in our study. The Taiwan Health Promotion Administration has adopted IOM guidelines (12.5–18 kg for underweight BMI < 18.5; 11.5–16 kg for 18.5 < normal BMI < 25; 7–11.5 kg for 25 < overweight BMI < 30; and 5–9 kg for obesity BMI < 30) while retaining Taiwan’s standard of 10–14 kg in the maternal medical instruction booklet [19,41]. In this study, women with underweight and normal pre-pregnancy BMIs had a significantly higher probability of delivering an infant with LBW compared to the other two BMI groups. These findings are similar to those of Waits et al. (2021), who used a nationally representative sample in Taiwan. If the mother gained 16–18 kg during the pregnancy period, she would not be the recommended range according to the Taiwan Health Promotion Administration. Further evidence is needed to develop Taiwanese local GWG guidelines to decrease confusion among pregnant women and healthcare providers [42]. Several epidemiologic studies have consistently shown a linear and direct relationship between GWG and fetal growth and birth weight [43] and that Western diets consisting of sweets and fast food were associated with excessive GWG [44]. However, our study found that in the “processed” group, almost 55% had “insufficient” GWG. We hypothesized that women with a processed dietary pattern would demonstrate more suboptimal knowledge and unhealthy behaviors, thus decreasing GWG associated with LBW. Such behaviors were defined as altering the food’s natural state (e.g., eating blanched, cooked, canned, frozen, dried, dehydrated, mixed, and packaged food) and consuming foods high in preservatives, flavors, additives, and substances (e.g., salt, sugars, and fats) [7]. In China, 151 (46.2%) women reported insufficient GWG of 15.2–22.6% [16,45]. Similarly, a nationwide survey in Taiwan in 2016 demonstrated that 41.5% of women had insufficient GWG [41]. This finding is consistent with the well-established link between GWG and low birth weight at term, both of which were independently influenced by a processed maternal diet in our study.

However, the participants in our study indicated that the media encouraged pregnant women not to get fat, and doctors in the prenatal clinic often stated that additional body weight can increase birth complications. Their responses are supported by a Taiwan-based study that demonstrated pregnant women are often conflicted about weight gain, following traditions, and esthetic concerns related to body image [46]. A qualitative inquiry among Asian women revealed adherence to social expectations of body size not only during pregnancy but also throughout their adult life [47]. Future studies measuring GWG and LBW should consider more dimensions, such as social environment and medical factors.

Researchers found that there is a greater prevalence of LBW in Taiwan (8.9%) than in China (4.9%), Australia (6.5%), and the United States (8%) [14]. An earlier study found that the prevalence of LBW in Taiwan increased from 5.3% to 7.0% during 2011–2016 [41]. This high prevalence suggests a need for routine assessments and proper care to improve the quality of dietary care during pregnancy. Compared with younger women, older pregnant women had a higher level of education and were full-time employees, and their families had a higher socioeconomic status. In Taiwan, we need to encourage age-appropriate information on pregnancy and childbirth. A further study is needed to identify factors associated with a higher risk of LBW among older women.

The present study has some limitations. First, this study was limited by its cross-sectional design. Thus, temporal and causal relationships could not be established. In addition, we enrolled participants from a web-based platform and did not have information on those who could not be approached, enhancing the possibility of participation bias. Second, food quantity and portion size were not documented in the questions on the food items. The lack of a daily analysis of the calorie content of meals and the energy expenditure of pregnant women is another limitation of this study. The quantities recorded for the food items are also prone to bias [8] because individuals generally do not describe food portions accurately, and there are substantial within-subject variations in the indication of food quantities [48]. Instead, a simpler list of food items for the FFQ is suggested and should be sufficient to indicate actual intakes [45]. Previous studies reported a positive association between the frequency of consumption and the actual intake of most foods [3,45]. Second, the self-reported data on pre-pregnancy weight could potentially introduce bias to the findings. Third, we measured dietary intake once, one month before delivery. It is a short period, and the patterns derived may not reflect participants’ diet in every stage of their pregnancy. Nevertheless, recent studies have suggested that overall dietary habits tend to remain stable throughout pregnancy [48,49]. Last, in this study, factor analysis with the principal component technique was adopted to identify dietary consumption patterns. This method is not without criticism, mainly due to the subjectivity in determining the number of factors to be extracted. This restriction has been minimized with the adoption of methodological assumptions in the statistical model [8].

## 5. Conclusions

The results of this study indicate that processed food consumption in the third trimester has negative effects on birth weight. We thus postulate that healthy food consumption can reduce unfavorable results and risks during pregnancy and childbirth for both the mother and the fetus. 

Food consumption, in general, and maternal diet, in particular, are modifiable. However, unsuitable modifications to maternal diet detrimentally impact the nutritional health of mothers and their infants, resulting in inappropriate GWG. Dietary and nutritional interventions during gestation, which is a key determinant of birth weight, may positively influence the health of the mother and fetus [49,50]. A primary factor contributing to LBW in Taiwan is insufficient GWG. Prenatal advice regarding GWG should be individualized. Importantly, emphasis should be placed on counseling and assisting pregnant women and prospective mothers to practice reducing unhealthy dietary intake (e.g., French fries, fried chicken, and pickled food). Nutritional counseling in antenatal clinics should focus on older and pre-pregnant women who are underweight.

## Figures and Tables

**Table 1:** Dietary pattern loading factors from factor analysis using varimax rotation.

Variables	Healthy	Processed
% of variance explained	25.28%	18.59%
Rice/noodles	0.71	
Bread and pasta/cereals	0.63	
Eggs	0.71	
Beef	0.59	
Mutton	0.45	
Pork	0.80	
Poultry	0.83	
Fresh fish	0.63	
Fresh seafood	0.66	
Beans/tofu	0.58	
Milk/dairy products	0.60	
Vegetables	0.69	
Pickled vegetables		0.68
fresh fruit	0.56	
Juice		0.50
Sugary drink		0.70
Tea with sugar		0.72
Soft drink		0.48
Chocolate products		0.60
Dessert		0.49
Pickled plum/sour plum		0.60
Fried chick/pried cutlet/French fries		0.79
Stir-fry and baste food		0.68
Fast food		0.77
Snacks/cakes/biscuits		0.68
Pickled food		0.70

**Table 2:** Characteristics of the study participants (N = 327).

	Overall Sample	Non-Advanced n = 151 n (%)	Advanced Age n = 176 n (%)	χ^2^	*p*-Value
Family socioeconomic status				5.00	0.08
High	153	61 (40.4)	92 (52.3)		
Middle	146	74 (49.0)	72 (40.9)		
Low	28	16 (10.6)	12 (6.8)		
Education level				3.57	0.17
High school or below	16	9 (6.0)	7 (4.0)		
University school	238	115 (76.2)	123 (69.9)		
Graduate school	73	27 (17.9)	46 (26.1)		
Work status				7.57	0.02 *
Full-time	180	73 (48.3)	107 (60.8)		
Part-time	38	16 (10.6)	22 (12.5)		
Housewife	109	62 (41.1)	47 (26.7)		
Pre-pregnancy BMI (kg/m^2^)				3.55	0.31
Underweight	45	25 (16.6)	20 (11.4)		
Normal	233	100 (66.2)	133 (75.6)		
Overweight	40	21 (13.9)	19 (10.8)		
Obesity	9	5 (3.3)	4 (2.3)		
Childbirth method				1.62	0.23 ^a^
Vaginal birth	229	111 (73.5)	118 (67.0)		
Cesarean birth	98	40 (26.5)	58 (33.0)		
GWG				2.02	0.37
Adequate	137	63 (41.7)	74 (42.0)		
Insufficient	151	66 (43.7)	85 (48.3)		
Excessive	39	22 (14.6)	17 (9.7)		
Pregnancy dietary pattern				0.45	0.50
Healthy	265	120 (79.5)	146 (82.4)		
Processed	62	31 (20.5)	31 (17.6)		
Infant sex				1.75	0.22 ^a^
Male	171	73 (48.3)	98 (55.7)		
Female	156	78 (23.9)	78 (23.9)		
Low infant birth weight				8.32	0.001 ^a^ *
No	298	145 (96.0)	153 (86.9)		
Yes	29	6 (4.0)	23 (13.1)		

Note: * *p* value < 0.05; ^a^: Fisher’s test.

**Table 3:** Dietary pattern of maternal characteristics (N = 327).

	Healthy Pattern (n = 265)	Processed Pattern (n = 62)	χ^2^	*p*-Value
Family socioeconomic status			2.22	0.33
High	129 (48.7)	24 (38.7)		
Middle	115 (43.4)	31 (50.0)		
Low	21 (7.9)	7 (11.3)		
Education level			1.70	0.43
High school or below	11 (4.2)	5 (8.1)		
University school	195 (73.6)	43 (69.4)		
Graduate school	59 (22.3)	14 (22.6)		
Work status			5.40	0.07
Full-time	152 (57.4)	28 (45.2)		
Part-time	26 (9.8)	12 (19.4)		
Housewife	87 (32.8)	22 (35.5)		
Pre-pregnancy BMI (kg/m^2^)			3.43	0.33
Underweight	36 (13.6)	9 (14.5)		
Normal	187 (70.6)	46 (74.2)		
Overweight	36 (13.6)	4 (6.5)		
Obesity	6 (2.3)	3 (4.8)		
Childbirth method			0.03	1.00 ^a^
Vaginal birth	185 (69.8)	44 (71.0)		
Cesarean birth	80 (30.2)	18 (29.0)		
GWG			5.40	0.07
Adequate	119 (44.9)	18 (29.0)		
Insufficient	117 (44.2)	34 (54.8)		
Excessive	29 (10.9)	10 (16.1)		
Low infant birth weight			17.16	<0.001 ^a^ **
No	252 (95.1)	46 (74.2)		
Yes	13 (4.9)	16 (25.8)		

Note: ** *p* value < 0.001. ^a^: Fisher’s test.

**Table 4:** Multiple logistic regression model for low birth weights (N = 327).

Variable	Crudes OR	95% CI	*p*-Values	Adjust OR	95% CI	*p*-Values
Maternal age						
<34 years	1			1		
≥35 years	3.6	1.4–9.2	0.01 *	5.8	2.0–16.6	0.001 *
Pre-pregnancy BMI						
Normal	1			1		
Underweight	3.3	1.4–8.1	0.01 *	6.8	2.3–20.1	0.001 *
Overweight/obese	1.1	0.4–3.5	0.85	2.1	0.6V7.9	0.28
GWG						
Adequate	1			1		
Insufficient	3.5	1.4–9.0	0.01 *	4.0	1.4–11.6	0.01 *
Excessive	1.2	0.2–6.1	0.84	0.9	0.1–5.5	0.88
Dietary pattern						
Healthy	1			1		
Processed	6.7	3.0–15.0	<0.001 **	9.4	3.7–23.6	< 0.001 **

Note: * *p* value < 0.05; ** *p* value < 0.001. The model after adjusting for family socioeconomic status, education level, and childbirth weeks.

## Data Availability

The data that support the findings of this study are available from the first author, upon reasonable request.
